# The influence of a scene preview on eye movement behavior in natural scenes

**DOI:** 10.3758/s13423-016-1035-4

**Published:** 2016-04-12

**Authors:** Nicola C. Anderson, Mieke Donk, Martijn Meeter

**Affiliations:** 1 Department of Cognitive Psychology, VU University Amsterdam, Van der Boechorststraat 1, 1081 BT Amsterdam, The Netherlands; 2 Department of Education Science, VU University Amsterdam, Amsterdam, The Netherlands

**Keywords:** Attention, Eye movements, Salience, Natural scene viewing, Contextual information

## Abstract

Rich contextual and semantic information can be extracted from only a brief presentation of a natural scene. This is presumed to be activated quickly enough to guide initial eye movements into a scene. However, early, short-latency eye movements in natural scenes have been shown to be dependent on the salience distribution across the image (Anderson, Ort, Kruijne, Meeter, & Donk, 2015). In the present work, we manipulated the salience distribution across a natural scene by changing the global contrast. We showed participants a brief real or nonsense preview of the scene and examined the time-course of eye movement guidance. A real preview decreased the latency and increased the amplitude of initial saccades into the image, suggesting that the preview allowed observers to obtain additional contextual information that would otherwise not be available. However, the preview did not completely override the initial tendency for short-latency saccades to be guided by the underlying salience distribution of the image. We discuss these findings in the context of oculomotor selection based on the integration of contextual information and low-level features in a natural scene.

## Introduction

A key question in research on oculomotor behavior in natural scenes is how such eye movements are controlled. It is generally accepted that our eyes and attention can be influenced by both the stimulus itself and by more cognitive factors, such as knowledge and task goals. In research utilizing simple displays, stimulus features capture attention even when participants have a strong top-down goal (Godijn & Theeuwes, 2002; Hunt, von Muhlenen, & Kingstone, 2007; Siebold, van Zoest, & Donk, 2011; Zehetleitner, Koch, Goschy, & Muller, 2013). In research utilizing natural scenes, such bottom-up effects on attention have been studied by comparing fixated locations to a salience map, which quantifies the relative conspicuity of individual features in the visual field (Foulsham & Underwood, 2008; Itti & Koch, 2000; Koch & Ullman, 1985; Mannan, Ruddock, & Wooding, 1996; Peters, Iyer, Itti, & Koch, 2005; Reinagel & Zador, 1999). Even though this represents a straightforward approach, the observed correlations also may be accounted for by more cognitive influences, such as the participants’ task (Anderson et al., 2015; Castelhano, Mack, & Henderson, 2009; Einhauser, Rutishauser, & Koch, 2008; Yarbus, 1967), the presence of a bias to look in the center of an image (Tatler, 2007), the meaning of the scene (Foulsham & Underwood, 2011), or the correspondence between objects and salience (Einhäuser, Spain, & Perona, 2008; Nuthmann & Henderson, 2010).

To estimate how salience affects natural scene viewing behavior, we recently performed a study in which we manipulated the salience distribution across an image (Anderson et al., 2015; see also Einhauser et al., 2008). We asked participants either to memorize a scene or to search for a bull’s-eye-shaped target. Critically, half of the scene was either reduced or increased in contrast relative to the other half, changing the overall distribution of salient regions across the image without the confounding factors that have bedevilled other attempts to correlate salience with eye movement behavior (Einhäuser, Rutishauser, & Koch, 2008). We found that this contrast manipulation influenced where participants attended. When they initiated their first saccade into the scene quickly, within approximately 300 ms after the onset of the image, they were more likely to land on the region of higher contrast. Beyond 300 ms after the presentation of the image, however, participants were almost equally likely to go to either side of the image. These results suggest that while long-latency and subsequent saccades might be based on more goal-driven or cognitive influences, short-latency eye movements are salience-driven. However, salience may not necessarily be the only driving force behind rapid initial selection.

Unlike synthetic, uniform displays that enforce tight control on salience and object placement, natural scenes have complex spatial arrangements that are rich in meaning. The visual system has much practice in extracting contextual information, semantics and objects from pictures of the real world. Indeed, a lot of information can be gleaned from just a brief glance (50-250 ms) at a scene. It can rapidly provide information about a scenes’ structure (Joubert, Rousselet, Fize, & Fabre-Thorpe, 2007) and semantics (Greene & Oliva, 2009). It is enough to establish the gist of a scene (Oliva & Torralba, 2006), its consistency (Davenport & Potter, 2004), and the presence and identity of some objects, people and animals (Fei-Fei, Iyer, Koch, & Perona, 2007; Thorpe, Fize, & Marlot, 1996). What is less well known, however, is to what extent such information influences eye movements occurring rapidly after the presentation of an image.

Torralba, Oliva, Castelhano, and Henderson (2006) proposed that both salience and contextual information are computed in parallel and integrated early on, before the occurrence of a first eye movement. Local (salience) and global (context) features are assumed to quickly converge into a contextually modulated salience map that may potentially affect even the fastest eye movements. Castelhano and Henderson (2007) and Vo and Henderson (2010) demonstrated that a briefly presented scene preview (as short as 50 ms), in conjunction with a prolonged delay between the preview and final image, reduced the time and the number of eye movements before a target was found. Already the first saccade into the scene was altered by the preview: its latency was reduced and its amplitude increased compared with a control condition without preview. This suggests that the preview allowed observers to extract contextual information that would otherwise not be available, which would run counter to the idea of Torralba et al. (2006) that contextual information is rapidly available. However, the scene preview also may have provided time for target knowledge to be integrated with the contextual representation extracted from the preview allowing subsequent eye movements to be quickly guided towards the target (Vo & Henderson, 2010). This would bring the results in line with the contextual guidance model of Torralba et al. (2006): a preview may not necessarily yield contextual information that would otherwise be missing but may allow a faster integration of that information with target knowledge. We sought to differentiate between these two possibilities by investigating whether a preview would also affect the first saccade into an image if there was no explicit top-down search goal and thus no need for any target-context integration.

In the present work, we presented participants with a brief preview of a scene that was either a normal preview of the upcoming image, or a nonsense image. After a delay, sufficiently long to establish a strong contextual representation, the final image was presented. Unlike the normal preview, the final image was manipulated to be reduced in contrast on one side of the image. The scene preview was shown long enough, and with a significant delay before the final scene onset to establish a strong representation of any gist or conceptual or semantic scene knowledge (Vo & Henderson, 2010). For convenience, we will refer to any information gleaned from the scene preview to be “contextual,” although see Wu, Wick, and Pomplun (2014) for an excellent discussion of the different forms of semantic information this definition might entail. The task for participants was to remember the scene for a later memory test to allow for relatively free exploration of the images.

If contextual information is available early enough to guide even the fastest first eye movements in a scene (Torralba et al., 2006), the preview type should not further affect initial selection behavior for there is no need for any target-context integration in the present set-up. Accordingly, the contrast distribution of the final image should affect initial eye movements equally in the normal relative to the nonsense preview condition. Alternatively, if contextual information is more gradually acquired, then a real preview should reduce the impact of the contrast distribution in the final image relative to a nonsense preview. This would be the case because a normal preview should then lead to a change in the contextually modulated salience map, such that the relative influence of context becomes larger at the expense of salience. This should lead to a reduced salience effect in the normal preview relative to the nonsense preview condition.

## Methods

### Participants

Sixteen participants (ages 18-28 years, *M* = 21.9 years, 94% female) were recruited from VU University Amsterdam and participated in this experiment for course credit or 9 Euros. All reported normal or corrected to normal vision and were naive to the purpose of the experiment. The study was approved by the ethics board of the Faculty of Psychology and Education and conducted according to the principles of the Declaration of Helsinki.

### Apparatus

The experiment was designed and presented using OpenSesame (Mathot, Schreij, & Theeuwes, 2012), an open source experiment programming environment integrated with the SR Research Eyelink 1000 tracking system (SR Research Ltd., Mississauga, Ontario, Canada). Stimuli were presented on a 22-inch (diagonal) Samsung Syncmaster 2233RZ with a resolution 1,680 × 1,050 pixels and refresh rate of 120 Hz at a viewing distance of 75 cm. Eye position was recorded via a second computer at 1,000 Hz with a spatial resolution of 0.01° visual angle using a 9-point calibration and validation procedure. The eye with the best spatial accuracy as determined by the calibration procedure was chosen for tracking. The online saccade detector of the eye tracker was set to detect saccades with amplitude of at least 0.5°, using an acceleration threshold of 9,500°/s^2^ and a velocity threshold of 35°/s. The experiments took place in a dim, sound-attenuated room. The experimenter received real-time feedback on system accuracy on a second monitor located in an adjacent room and calibration and validation was repeated as needed.

### Stimuli

Images were selected from the SUN2012 Database (Xiao, Hays, Ehinger, Oliva, & Torralba, 2010) and from “Learning to Predict where Humans Look” (Judd, Ehinger, Durand, & Torralba, 2009). The images depicted various exteriors, interiors and natural scenes and were chosen such that they did not contain any obvious human faces or text. Each image had a native resolution of 1,024 × 768 pixels and was converted to greyscale. Images were further selected such that their mean intensity values across the left and right side of the image conformed to a ratio of at most 3:4. Conforming to these selection criteria, 100 images were used from the SUN2012 Database and 91 images from “Learning to Predict where Humans Look.” Stimuli were presented centrally on the monitor at their native resolution and subtended a visual angle of approximately 22 degrees horizontal by 16 degrees vertical. The rest of the screen surrounding the image was gray.

### Contrast adjustment

**Fig. 1:**
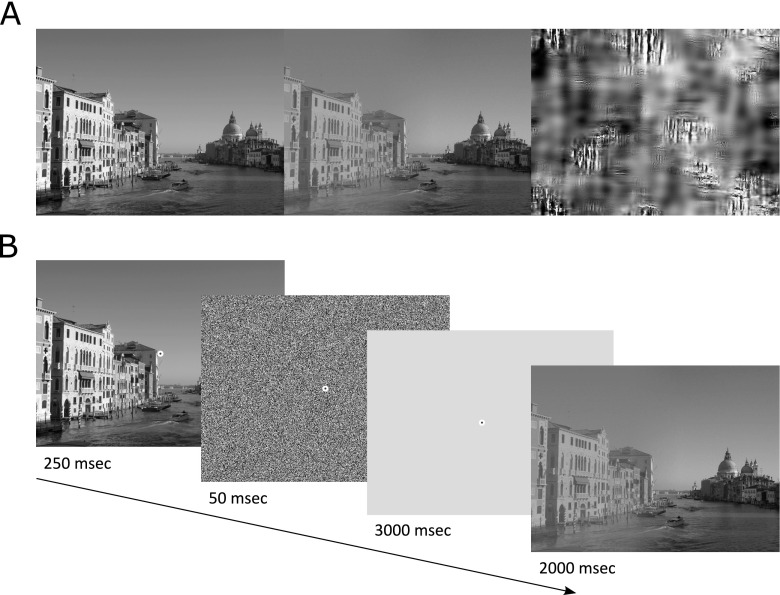
**a** Example of an original grayscale image, the same image with reduced contrast on the left and the synthesized scrambled “nonsense” preview. **b** Schematic representation of an encoding trial with a normal preview

Image contrast adjustment was performed on all selected images using Matlab’s imadjust function (MATLAB, 2011). Intensity values on one side of the image were linearly remapped to a range spanning 40% of the original intensity range, in a way that left mean intensity unaffected. Only the variance in intensity was thus reduced. For each image, either the left or right 2/5 of the image width was manipulated in this fashion. For the center 1/5, contrast was reduced gradually, from full to reduced contrast, leaving the remaining 2/5 of the image width unmanipulated. Two versions of each image were created, either with the contrast gradually reduced from left to right or from right to left (Fig. 1a). Salience maps were computed for each modified image using the Saliency Toolbox (Walther & Koch, 2006). Mean salience was significantly lower for the reduced contrast side of each image (*M* = 0.005) compared with the original (*M* = 0.03), *t*(381) = 38.59, *p* < 0.001.

### Scrambled images

A “scrambled” version of each image was created using a texture synthesis algorithm created by Portilla and Simoncelli (2000) but modified by Greene and Oliva (2009). The algorithm takes the image as input and calculates a number of image statistics, such as orientation and luminance information. It then coerces a noise stimulus to have the same properties. The resulting stimuli have similar low level perceptual features as the scene input stimulus, but without any object or spatial layout information (Greene & Oliva, 2009).

### Procedure

Participants were seated with their head constrained in a chin rest and were given verbal and written instructions regarding the experimental procedure. Calibration and validation of their eye position was performed. In the first phase of the experiment, participants were instructed to explore the images carefully to remember the images for a later recognition task. Each trial began with a drift-correction screen in which participants were required to press the spacebar while fixating a centrally presented circular dot. Participants were then given either a preview of the image that they were about to see that was an unmanipulated version (i.e., without the contrast adjustment) of the final image, or a nonsense preview that was a scrambled version of the image (Fig. 1a). The preview or nonsense image was presented for 250 ms immediately followed by a 50-ms noise mask, then a 3-s uniform grey “integration” screen. The image was then presented for 2 s (Fig. 1b). The previews and integration screens contained a centrally presented circular fixation dot and participants were instructed to fixate on the dot for as long as it appeared onscreen. This dot disappeared when the final image was presented and participants were then free to move their eyes throughout the image. Figure 1b depicts a possible trial sequence.

**Fig. 2:**
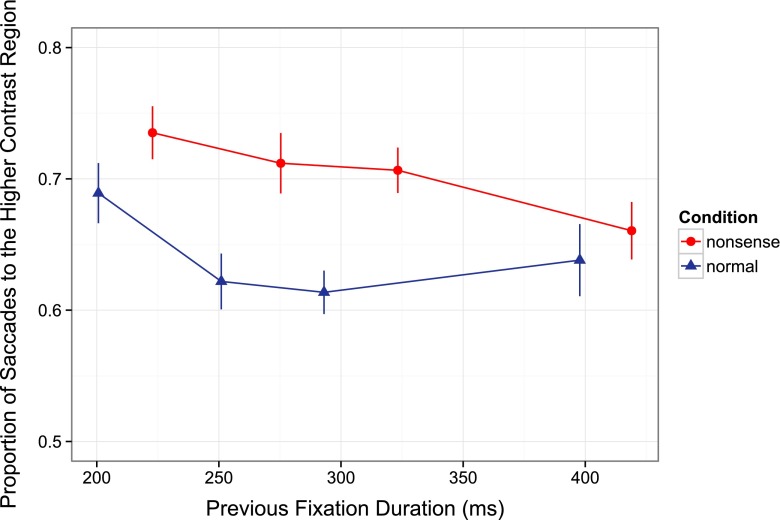
Proportion of saccades that landed in the higher contrast region of the image separately for each preview condition and saccade latency bin. Error bars in this and all subsequent figures represent standard error corrected for between-subjects variance (Cousineau, 2005; Morey, 2008)

We selected 150 images from the dataset, with the contrast adjusted on both the left and right. These images were presented in 300 trials with a mixed random order such that each participant saw the same image (with a different side adjusted) twice throughout the first phase of the experiment, with the restriction that participants could never see the second version of the image within the following three consecutive trials. The images were presented in 10 blocks and after each block participants were given feedback about their progress through the experiment.

At the end of the experiment, participants performed a recognition task on 20 images that had been presented in the initial phase and 20 new images (these images were randomly chosen from the image pool and were a mix of image categories). Each image was presented for 2 s, after which participants were asked to press the “z” key if they had seen the image before, or the “/” key if they had not seen the image before.

To familiarize participants with the task, participants performed 14 practice trials followed by 14 recognition trials (7 “old” images and 7 “new” images) before the experimental phase. Immediately after the practice trials, participants were given feedback on their performance in the recognition phase and had an opportunity to ask questions about the experimental procedure. At the conclusion of the experiment, participants were given feedback on their performance in the recognition task and then were asked to type in a field on the screen whether or not they had “noticed anything strange about the images.” This was done to check whether the contrast adjustment was noticed by the participants. The entire experiment lasted approximately 60 minutes.

### Data processing

Fixations were removed if their duration was longer than 700 ms or faster than 120 ms or if they started outside a 65 pixel radius (approximately 1 degree visual angle) from the central fixation dot. These restrictions resulted in the removal of 2.6% of the trials.

The primary dependent measure was the proportion of initial saccades that landed in the higher contrast region. This region was either the left or right side of the image, from the center to the edge of the image boundary (i.e., including half of the middle 20% of the image that was gradually reduced in contrast at one side). A second dependent measure, saccade amplitude (in degrees visual angle) was calculated to assess the potential impact of the various preview types on saccadic targeting. A third measure analyzed was the latency of the initial saccade, calculated from the onset of the final image. We focus only on the first saccade into a scene for two reasons. First, it has been demonstrated previously that these eye movements are influenced by the contrast manipulation, whereas subsequent saccades are less affected by it (Anderson et al., 2015). Second, because we do not employ a gaze-contingent design when participants view the final image, we cannot make inferences about how any contextual representation built during the scene preview influences these eye movements.

## Results

### Performance

Seven out of the 16 participants indicated in the questionnaire that they had noticed the contrast manipulation.^1^ Recognition accuracy was 91.6% (*SE* = 1.74%).

### Proportion of eye movements to the higher contrast region

Figure 2 shows the proportion of saccades that landed on the higher contrast region of the image as a function of their saccade latency for both the normal and nonsense preview conditions. A 2 (preview type: nonsense vs. normal) by 4 (saccade latency bin) within-subjects analysis of variance was conducted on the proportion of first saccades that landed on the higher contrast region of the image, with the saccade latency factor treated as a linear contrast.

There was a main effect of preview type, *F*(1, 15) = 30.91, *MSE* = 0.008, *p* < 0.001, ${\eta}_P^2$ = 0.673, such that saccades landed more often on the higher contrast region when the preview was a nonsense image compared with when it was a normal image. There was a marginal main effect of saccade latency bin, *F*(1, 15) = 3.85, *MSE* = 0.009, *p* = 0.069, ${\eta}_P^2$ = 0.204, such that short-latency first saccades tended to land more often on the higher contrast side of the image than long-latency saccades. There was no interaction between preview type and saccade latency bin, *F*(1, 15) = 0.34, *MSE* = 0.010, *p* = 0.569, ${\eta}_P^2$ = 0.022.

Figure 2 suggests that when the preview was a normal image, selection of the higher contrast region may vary with latency in a non-linear fashion. To investigate the time course of selection performance across conditions, we performed a follow-up analysis of variance with saccade latency bin as a quadratic factor (Fig. 2). There was no quadratic main effect of bin, *F*(1, 15) = 1.43, *MSE* = 0.004, *p* = 0.250, ${\eta}_P^2$ = 0.087, but there was a significant interaction between preview type and latency bin, *F*(1, 15) = 8.64, *MSE* = 0.006, *p* = 0.010, ${\eta}_P^2$ = 0.364. This interaction likely results from the particular tendency, when the preview was a normal image, for the shortest-latency saccades (from the first saccade latency quartile; Fig. 2) more often to land on the higher contrast region of the image than those from later latency quartiles. However, for each bin and across both normal and nonsense preview types, first saccades were more likely than chance to land on the higher contrast region, all *t* > 4.87, *p* < 0.001.

### Saccadic amplitude

**Fig. 3:**
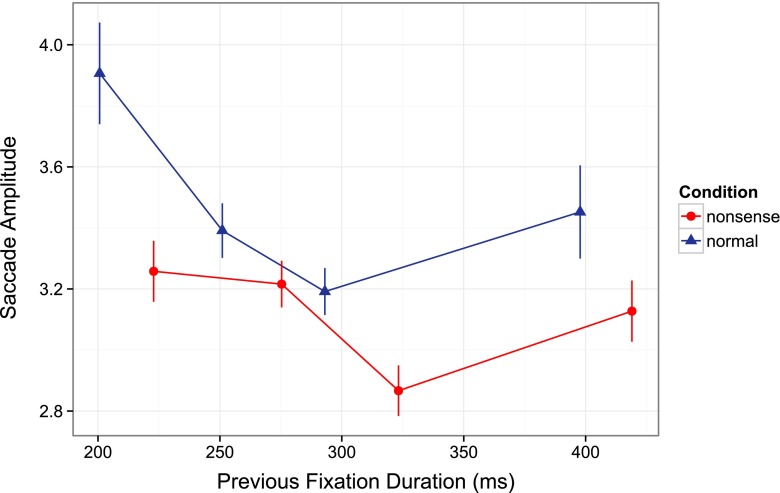
Saccade amplitude separately for each preview condition and saccade latency bin

Figure 3 shows the amplitude of the first saccade as a function of its latency for both preview conditions. A 2 (preview type: nonsense vs. normal) by 4 (saccade latency bin) within-subjects analysis of variance was conducted on the first saccadic amplitude, with saccadic latency factor treated as a linear contrast.

There was a main effect of preview type, *F*(1, 15) = 35.78, *MSE* = 0.222, *p* < 0.001, ${\eta}_P^2$ = 0.705, such that saccade amplitude was significantly larger when the preview was a normal image, compared with when the preview was a nonsense image. There was no linear effect of saccade latency bin, *F*(1, 15) = 3.02, *MSE* = 0.348, *p* = 0.013, ${\eta}_P^2$ = 0.168, and no interaction between preview type and saccade latency bin, *F*(1, 15) = 2.52, *MSE* = 0.192, *p* = 0.133, ${\eta}_P^2$ = 0.144.

We also performed a follow-up analysis of variance with saccade latency bin as a quadratic factor on the first saccade amplitude. There was a quadratic main effect of latency bin, *F*(1, 15) = 17.87, *MSE* = 0.060, *p* = 0.001, ${\eta}_P^2$ = 0.544, but no interaction between preview type and saccade latency bin, *F*(1, 15) = 2.74, *MSE* = 0.332, *p* = 0.119, ${\eta}_P^2$ = 0.154.

### Latency of the first saccade

The latency of the first saccade into the image was significantly shorter when the preview was a normal image (*M* = 286 ms; *SD* = 25.21 ms) than when the preview was a nonsense image (*M* = 310 ms; *SD* = 26.59 ms), *t*(15) = 6.57, *p* < 0.001.

## Discussion

The results of the present work revealed that a brief preview of an image was enough to influence saccadic programming to the extent that the tendency to move the eyes to the higher-contrast region was reduced relative to when the preview was a nonsense image. A preview of a natural scene significantly shortened the latency and increased the amplitude of the initial saccade. This finding extends previous work (Vo & Henderson, 2010) by demonstrating that a preview influences short-latency initial saccades into a scene. The present work additionally shows that this occurs even when there is no subsequent disruption to scene context (Vo & Henderson, 2010). More importantly, the results demonstrate that a preview affects initial saccades while observers were not engaged in any search task. This suggests that a preview effect may not only arise because the preview prolongs the available context-target integration time (Vo & Henderson, 2010) but also because it enhances the relative influence of context on oculomotor selection behavior (at the expense of the contribution of salience).

Nevertheless, the scene preview was not enough to override completely an early tendency to look toward the region of the image with a higher contrast, indicating that salience, to some extent, still affected oculomotor selection behavior. This finding contrasts with work suggesting that a contextual representation built during a 250-ms image preview should allow selection to be completely guided in a top-down fashion (Brockmole & Henderson, 2008; Vo & Henderson, 2010), and even when not provided with an image preview, much work suggests that goal-driven processes can rapidly influence eye movement behavior (Henderson, Malcolm, & Schandl, 2009; Neider & Zelinsky, 2006; Spotorno, Malcolm, & Tatler, 2014; Torralba et al., 2006; Vo & Henderson, 2010).

Whereas the idea that stimulus salience may influence oculomotor control is against theories suggesting that selection behavior is driven predominantly by cognitive factors (Henderson, Brockmole, Castelhano, & Mack, 2007; Neider & Zelinsky, 2006; Spotorno, Malcolm, & Tatler, 2014), such earlier work has not investigated selection behavior on such a fine-grained timescale as in the present work (see also: Anderson et al., 2015; Mackay, Cerf, & Koch, 2012). Our results are in line with a view of oculomotor control that integrates both stimulus salience and knowledge structures (Navalpakkam & Itti, 2005; Torralba et al., 2006). Our findings here and in studies utilizing more simple displays (Donk & van Zoest, 2008; van Zoest & Donk, 2008; van Zoest, Donk, & Theeuwes, 2004) further constrain this relationship by suggesting that salience may be perceived as an emergent property of the speed at which individual objects are processed in the visual system. In this view, salience is coded in the temporal as well as spatial domain, where more conspicuous regions receive earlier activation than less conspicuous regions. This results in a selection bias for salient regions for early, fast responses, but eventually leads to a state of equivalence across locations that stand out from the background.

The present findings suggest that even though both salience and context determine selection behavior early on in scene viewing (Torralba et al., 2006), the relative contribution of context can be increased by the presentation of a real preview. This latter finding is in line with the idea that the contextual representation is acquired during a longer period of time than previously assumed (Torralba et al., 2006) and suggests that context, like salience exerts its influence through a dynamically changing representation in time.

## Conclusions

We demonstrated that a brief preview of a natural scene provides some contextual guidance of eye movements. It can decrease the latency and increase the amplitude of the first saccade into a natural scene. In addition, it reduces the tendency for initial saccades to be guided by the salience distribution of the scene. The contextual representation built during the preview, however, was not strong enough to completely override the influence of salience, as saccades, particularly those with the shortest latency, were more likely than chance to land on the higher contrast side of the image.

## Footnotes

1 When submitted as a between subjects factor, whether people noticed the manipulation did not influence the proportion of eye movements that landed on the higher contrast region, *F* < 1, nor did it interact with preview, *F* < 1, bin, *F*(1,14) = 1.54, *MSE =* .008, *p* = .234, ${\eta}_P^2$ = .099, nor the preview by bin interaction, *F* < 1. This was also the case for saccade amplitudes, all *F*’s < 1.
